# Contrastive Clustering-Based Patient Normalization to Improve Automated In Vivo Oral Cancer Diagnosis from Multispectral Autofluorescence Lifetime Images

**DOI:** 10.3390/cancers16234120

**Published:** 2024-12-09

**Authors:** Kayla Caughlin, Elvis Duran-Sierra, Shuna Cheng, Rodrigo Cuenca, Beena Ahmed, Jim Ji, Mathias Martinez, Moustafa Al-Khalil, Hussain Al-Enazi, Javier A. Jo, Carlos Busso

**Affiliations:** 1 Department of Electrical and Computer Engineering, The University of Texas at Dallas, Richardson, TX 75080, USA; 2 Department of Biomedical Engineering, Texas A&M University, College Station, TX 77840, USA; eduran3@mdanderson.org (E.D.-S.);; 3 School of Electrical and Computer Engineering, University of Oklahoma, Norman, OK 73019, USAjavierjo@ou.edu (J.A.J.); 4 School of Electrical Engineering and Telecommunications, University of New South Wales, Sydney, NSW 2033, Australia; 5 Department of Electrical and Computer Engineering, Texas A&M University at Qatar, Doha 23874, Qatar; 6 Department of Cranio-Maxillofacial Surgery, Hamad Medical Corporation, Doha 3050, Qatarmalkhalil@hamad.qa (M.A.-K.); 7 Department of Otorhinolaryngology Head and Neck Surgery, Hamad Medical Corporation, Doha 3050, Qatar; 8 Language Technologies Institute, Carnegie Mellon University, Pittsburgh, PA 15213, USA

**Keywords:** multispectral autofluorescence lifetime imaging, automated cancer diagnosis, margin delineation, patient normalization, regularization, deep learning

## Abstract

**Background:** Multispectral autofluorescence lifetime imaging systems have recently been developed to quickly and non-invasively assess tissue properties for applications in oral cancer diagnosis. As a non-traditional imaging modality, the autofluorescence signal collected from the system cannot be directly visually assessed by a clinician and a model is needed to generate a diagnosis for each image. However, training a deep learning model from scratch on small multispectral autofluorescence datasets can fail due to inter-patient variability, poor initialization, and overfitting. **Methods:** We propose a contrastive-based pre-training approach that teaches the network to perform patient normalization without requiring a direct comparison to a reference sample. We then use the contrastive pre-trained encoder as a favorable initialization for classification. To train the classifiers, we efficiently use available data and reduce overfitting through a multitask framework with margin delineation and cancer diagnosis tasks. We evaluate the model over 67 patients using 10-fold cross-validation and evaluate significance using paired, one-tailed *t*-tests. **Results:** The proposed approach achieves a sensitivity of 82.08% and specificity of 75.92% on the cancer diagnosis task with a sensitivity of 91.83% and specificity of 79.31% for margin delineation as an auxiliary task. In comparison to existing approaches, our method significantly outperforms a *support vector machine* (SVM) implemented with either *sequential feature selection* (SFS) (*p* = 0.0261) or L1 loss (*p* = 0.0452) when considering the average of sensitivity and specificity. Specifically, the proposed approach increases performance by 2.75% compared to the L1 model and 4.87% compared to the SFS model. In addition, there is a significant increase in specificity of 8.34% compared to the baseline autoencoder model (*p* = 0.0070). **Conclusions:** Our method effectively trains deep learning models for small data applications when existing, large pre-trained models are not suitable for fine-tuning. While we designed the network for a specific imaging modality, we report the development process so that the insights gained can be applied to address similar challenges in other non-traditional imaging modalities. A key contribution of this paper is a neural network framework for multi-spectral fluorescence lifetime-based tissue discrimination that performs patient normalization without requiring a reference (healthy) sample from each patient at test time.

## Simple Summary

Lip and oral cavity cancer caused over 177,000 deaths globally in 2020, but patient survival increases with earlier diagnosis. One barrier to early diagnosis is the invasive nature of biopsies needed for diagnosis. Automated diagnosis systems have the potential to perform non-invasive diagnosis by pairing novel imaging data with deep learning models. Given the variability between patients, access to a sufficiently large training database from human subjects limits deep learning applications. We propose a model that maps non-invasive images of oral tissue to a diagnosis by encouraging the model to group normal samples close together (reducing variability between patients). Our model improves non-invasive oral cancer diagnosis through a robust training process that only requires a small amount of data. This work shows how we can address small data challenges through model architecture and training, rather than through the collection of larger databases or manual corrections and normalizations.

## 1. Introduction

In 2020, lip and oral cancer caused over 177,000 deaths globally [1]. In particular, countries with lower *human development indices* (HDIs) saw increased incidence and mortality rates for lip and oral cancer [1]. Efforts to develop automated oral cancer classifiers result from the clinical need to easily and reliably classify oral lesions, especially in low HDI regions. Both margin delineation (classification of lesion tissue versus healthy tissue) and diagnosis (classification of lesion tissue as benign or malignant) have clinical relevance. While diagnosis classifiers are useful for determining if treatment or resection is required, margin delineation classifiers are useful in image-guided surgery to ensure the margin of the lesion is removed during resection. We are interested in *multispectral fluorescence lifetime imaging* (maFLIM) since this imaging modality is fast and non-invasive. In contrast to whole-slide images and mobile camera images (RGB format), we note that maFLIM images in our domain provide in vivo metabolic and biochemical information in the form of three biexponential-like decays per pixel. As a result, commonly used medical image processing architectures such as U-Net [2] and networks pre-trained on ImageNet (e.g., [3,4,5]) cannot simply be fine-tuned for our application. Similarly, typical data augmentation methods such as scaling, flipping, or rotation do not apply to our domain.

A key challenge in developing an automated diagnosis or delineation classifier in maFLIM is the variability of oral lesions. Within lesions of the same class, tissue characteristics from different patients may differ due to the lesion location (e.g., tongue versus tonsil) [6]. In addition, oral cancers are associated with a variety of different risk factors, including *human papillomavirus* (HPV) and tobacco use [7]. To reduce inter-patient variability, some authors have proposed incorporating a reference (healthy) and suspicious (potentially malignant) example for each patient [8,9]. Adversarial methods have also been used to reduce inter-patient variability [10]. One patient normalization approach in our domain calculates features for the healthy and suspicious images and uses the difference of each feature’s healthy and suspicious values as the input to a classifier [8,9]. Outside of a reference value for patient normalization, the healthy samples are not typically used when training networks for oral cancer diagnosis [8,9]. Similarly, margin delineation approaches may only compare malignant and healthy samples, discarding the benign images [11,12].

**Figure 1:**
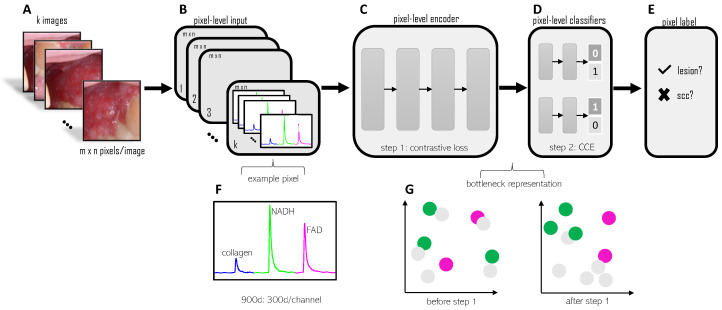
Approach overview. Step 1 (**A**–**C**)—input pixel-level training data (see example pixel panels (**B**,**F**)) into the encoder and train using contrastive loss. After step 1 pre-training, the bottleneck representation shows the clustering of each class (see bottleneck representation Panel (**G**)). Step 2 (**D**,**E**)—add pixel-level classifiers and train with categorical cross-entropy losses. Aggregate all pixel-level labels from a single image using a 50% threshold to label each image.

In this work, we explore two main questions: (1) Can we achieve a reduction in inter-patient variability without direct correspondence between healthy and suspicious images from each patient? (2) Can we combine the two clinically relevant tasks of margin delineation and cancer diagnosis in a beneficial way? Our method consists of two steps: contrastive pre-training followed by the addition of two task classifiers. Samples from three classes are used: benign, malignant, and healthy. The contrastive pre-training step trains an encoder that embeds each pixel such that pixels from the same class cluster together, away from pixels from a different class (see bottleneck representation in Figure 1). Through the contrastive pre-training, we force the encoder to focus on characteristics specific to the class, rather than specific to a particular patient. Instead of normalizing each lesion feature by subtracting a paired healthy feature value, our approach uses contrastive learning to create patient-invariant representations of each image, whether healthy or suspicious. Through our patient normalization, we reduce inter-patient variability without any direct pairing between a patient’s healthy and suspicious images. In addition to the contrastive pre-training, we add task classifiers for the margin delineation and diagnosis tasks. The multitask framework provides enough regularization to the network to avoid the use of an autoencoder as reported in Caughlin et al. [13] and allows both clinically relevant tasks to be accomplished with the same encoder.

Our approach improves diagnosis performance in comparison to competitive baselines. In addition, our network achieves good performance on the margin delineation task, even though delineation is treated as an auxiliary task within our framework. Our results show that training a multitask model can help regularize the training process for several clinically relevant tasks, while contrastive learning can reduce inter-patient variability (a key challenge in automated medical tasks). Our full cancer diagnosis model achieves a sensitivity of 82.08% and a specificity of 75.92%. The improvements over baselines range from 1.46% to 4.87% considering the average of sensitivity and specificity. In summary, the primary contributions of our work are implicit patient normalization using a contrastive pre-training step and further regularization through a novel multitask network for margin delineation and cancer diagnosis of oral lesions.

The rest of the paper is organized as follows: Section 2 describes related work in terms of maFLIM classification, contrastive learning, and multitask learning. Section 3 details the loss functions and training process. Section 4 analyzes the contrastive loss function and model performance, including baseline comparison and model analysis. Section 5 summarizes the results and suggests further research directions.

## 2. Related Work

### 2.1. Classification Approaches in maFLIM

Tasks for automated classification of oral lesions using maFLIM include margin delineation and cancer diagnosis. In margin delineation, the task is to distinguish between healthy tissue versus cancerous tissue. In cancer diagnosis, the task is to determine if a lesion is benign or cancerous. Each task is typically treated separately using a machine learning approach, with variations on lifetime and intensity features input into each classifier. For example, Duran et al. [11] evaluated *support vector machines* (SVMs), *quadratic discriminant analysis* (QDA), and ensemble approaches on the margin delineation task. They used only healthy, cancerous, and pre-cancerous training data, without incorporating any images from benign lesions, and did not consider the cancer diagnosis task. Jo et al. [8] used similar classifiers for the cancer diagnosis task, where the classifiers were trained on patient-normalized features from benign, cancerous, and pre-cancerous lesions. While healthy samples were not included as a separate class, the patient normalization step required each patient in both the training set and the testing set to have two images: one of the lesion and one of the healthy tissue [8]. Similarly in the skin cancer domain, Vasanthakumari et al. [9] used SVM, QDA, and *linear discriminant analysis* (LDA) classifiers trained with phasor features for cancer diagnosis of skin lesions.

Transitioning to deep learning approaches, Marsden et al. [6] implemented a pre-trained *convolutional neural network* (CNN) for the margin delineation task. In cancer diagnosis, we previously introduced an autoencoder and classifier approach [13]. However, our previous work only considered the benign and cancerous or pre-cancerous samples during training and applied no patient normalization. While our approach improved classification performance over machine learning baselines, the addition of patient normalization could increase classification performance. While patient normalization could be accomplished in a variety of ways, we focus on a contrastive pre-training step without direct correspondence between healthy and lesion images from the same patient.

### 2.2. Contrastive Learning Background

The primary objective of typical contrastive losses is to map the input to a space where the distances between samples are meaningful for a task [14,15,16,17]. The variations consist of differences in the number of positive and negative examples used (i.e., pairs, triplets, or N-pairs) or in the way that samples are grouped (i.e., from class labels, data augmentations, or temporal separation).

Considering the number of positives and negatives, Chopra et al. [18] described a loss function based on a Siamese network, where a sample was contrasted with either a positive or a negative example using the L1 distance. The model learned to map images of the same person into a space where the images are close together. Images of different people were mapped to a space where the distance between the images was large [18]. In addition to the positive pair in Chopra’s contrastive loss, the triplet loss also considers a negative example for each input [14,15]. The triplets consist of a sample (the anchor), an example from the same class as the anchor (the positive), and an example from a different class (the negative). Weinberger et al. [14] and Schroff et al. [15] used triplets of samples in a hinge-based loss. In the hinge loss approach, the model is penalized when the distance between two samples with different labels does not exceed the distance between two samples of the same label by a margin [14,16]. In Weinberger et al. [14], the triplets were formed using the *k* neighbors, and an additional term was added to cluster the anchor and positive example.

Following contrastive and triplet losses, Sohn [16] introduced the N-pair loss. The N-pair loss modifies the triplet loss to consider *N-1* negative samples for each anchor [16]. By using more negative examples, the loss is less dependent on hard negative mining, shows faster convergence, and leads to improved performance [16]. Moving further, Khosla et al. [17] introduced a loss function for contrastive pre-training using class labels. Their formulation uses the class labels of the samples in each batch to form positives and negatives, where each positive example in the batch and each negative example in the batch contributes to a sample’s loss. Using a two-stage training process, the results from Khosla et al. [17] showed that a contrastive pre-training step improves classification performance over a single cross-entropy classifier training stage, even though both steps are fully supervised.

In the medical imaging domain, various losses and pairs strategies have been explored [10,19,20,21]. Choudary et al. [20] used a triplet loss for *image quality assessment* (IQA) on whole slide images, starting with a pre-trained CNN. Contrastive learning using different images from the same patient has been used for X-ray images [21]. Similarly, augmentations of the same signal have been used for contrastive learning in *electroencephalogram* (EEG) and *electrocardiogram* (ECG) tasks [10]. Both of these frameworks used the InfoNCE loss [10,21]. Goswami et al. [19] used losses similar to ours to reduce inter-patient variability in leukemia classification. The authors trained on a dataset of images from microscopes, enabling the use of a standard CNN architecture pre-trained on ImageNet [19]. We note that raw maFLIM data are not compatible with pre-trained CNNs from natural images, so novel approaches are needed, especially with reduced training data.

### 2.3. Multitask Learning Background

Rather than training on a single task, multitask learning uses a shared hidden layer that connects to more than one task. Caruana [22] showed that training with more than one task simultaneously improves generalization and allows for the learning of different features. The specific formulation of these extra tasks varies; they could involve predicting features present in the training set but absent in the test set [22]. The extra tasks could also involve predicting future values [22]. In these formulations, the extra tasks are used solely to guide training [22]. Alternatively, all the training tasks could be utilized during both training and testing phases [22].

In our case, we used two related tasks: margin delineation and cancer diagnosis. In general, we found that the margin delineation (distinction between malignant versus healthy) is easier than the classification task (distinction between malignant versus benign). In a setting where some tasks are easier than others, Caruana hypothesized that the easier tasks can help identify relevant features that the more difficult task might struggle to learn [22]. The multitask framework can also learn features that neither task alone would identify [22]. Finally, multitask learning also uses small datasets efficiently because more information is available during training on multiple tasks (called “data amplification” by Caruana [22]).

In the bioimaging domain, various multitask formulations have led to improvements in automated cancer diagnosis [23,24,25]. Seo et al. [23] used multitask learning to automatically locate tumors in lung or liver *computed tomography* (CT) images. The authors train a CNN using as few as 48 images. They attributed the success of their method, in part, to the increased regularization of the network from multitask training [23]. Similarly, Khosravan and Bagci [24] trained a CNN on CT scans to find lung nodules and to determine if a proposed region is a lung nodule (“false positive reduction”). The authors emphasize the benefits of multitask learning for improved generalization, discriminative feature identification, and training on small data [24]. Sainz et al. [25] used a multitask network to diagnose breast cancer and to locate lesions and calcifications from mammograms.

While the same multitask learning insights apply to our research, we note that our imaging modality is completely different from CT or mammography. Common imaging modalities like CT and mammography show anatomical information and are typically processed by the network on an image level. In contrast, maFLIM data primarily shows functional information related to the tumor microenvironment. Furthermore, we input pixel-level information into our network. As shown in Figure 1 Panel F, each pixel contains a time-series sequence.

## 3. Materials and Methods

### 3.1. Motivation

Existing patient normalization approaches directly compare pairs of images from each patient: a healthy reference sample and a suspicious/lesion sample. In contrast, we want to incorporate patient normalization into the training process. The network will learn to remove patient-specific characteristics and identify common class-specific features across patients, without paired comparison of each patient to their reference sample. Ideally, the contrastive pre-training step organizes the feature space such that each class groups together far from the other classes (see bottleneck representation, Figure 1). Building on the success of multitask learning, we want to add classifiers that reduce overfitting and allow the primary and auxiliary tasks to benefit from each other, leveraging a favorable initialization from the pre-training step. We hypothesize that the contrastive plus multitask framework will reduce inter-patient variability, smooth the decision boundaries, and regularize the network to improve generalization *without requiring two samples from each patient at test time*.

### 3.2. Contrastive Pre-Training

As stated in a recent survey, “contrastive learning is an idea, not a specific model [26]”. While contrastive learning is often used to generate the representation of entire images, the concept is not exclusive to images. It has been used in other modalities as well, such as text summarization [27], graph data [28], and video [29]. We note that in contrastive learning with images, the loss is generally applied to the embedding (hidden layer) rather than directly to the image input [26]. Similarly, our encoder maps the 900-dimensional pixel input (containing three biexponential decays in a single pixel) to a lower-dimensional embedding where we can apply a contrastive loss. The contrastive loss reduces variability by bringing the reference (normal) pixels closer together in the embedding space. We can think of this process as a learned normalization. Fernando et al. [30] discussed how natural variability and measurement variability in patient data lead to challenges in medical anomaly detection, providing an example of patient endoscopy images that belong to separate classes but are visually similar. Similarly, in the presence of high inter-patient variability, the distance between an individual patient’s normal and lesion data may be smaller than the distance between two different patients’ normal images. In this setting, the model may reach a local optimum by overfitting to individual data points rather than learning generalizable characteristics. Using the contrastive loss, we guide the model to ignore patient-specific characteristics that are irrelevant to the task of cancer diagnosis. This contrastive pre-training creates a better decision boundary, where the model learns to focus on generalizable characteristics that differentiate cancer data from normal tissue data. This is conceptually similar to forcing the model to view perturbations of the same image as similar, where the perturbation here is the natural variability among patients of the same class.

The pre-training step starts with an encoder (see Figure 1), which takes in unpaired pixel-level data from three classes: healthy, benign, and malignant. We apply a contrastive loss on the encoder embedding to train the network to cluster distinct classes. We optimize the total contrastive loss, $L_{contr}$, which consists of *clustering* terms and *separation* terms:

(1) $$L_{contr}=L_{clust}-L_{sep}$$

where $L_{clust}$ is the clustering loss we seek to minimize and $L_{sep}$ is the separation loss we seek to maximize (hence, the negative sign in Equation (1)). We chose a summation of simple squared-error-based loss terms. While this strategy allows analysis of each loss term, we note that other contrastive losses may provide additional improvement. The clustering loss is given as follows:

(2) $$L_{clust}=\sum_{N_{c}}\alpha_{c}\sum_{N_{pospt}}{\left({\mathrm{Emb}}_{\mathrm{anchor}}-{\mathrm{Emb}}_{\mathrm{positive}}\right)}^{2}$$

$N_{c}$ refers to the number of classes (three in our case) and $N_{pospt}$ refers to the number of patients with the same class as the anchor. The anchor within a single class, ${\mathrm{Emb}}_{\mathrm{anchor}}$, is the mean embedding of the class within the batch. An anchor-positive pair is formed by taking the mean embedding of each same-class patient in the batch (${\mathrm{Emb}}_{\mathrm{positive}}$).

The separation loss is similar, but compares the anchor embedding to embeddings from the other classes:

(3) $$L_{sep}=\sum_{N_{c}}\beta_{c}\sum_{N_{negpt}}{\left({\mathrm{Emb}}_{\mathrm{anchor}}-{\mathrm{Emb}}_{\mathrm{negative}}\right)}^{2}$$

where ${\mathrm{Emb}}_{\mathrm{negative}}$ is the mean embedding of the negative class patient within the batch. $\alpha_{c}$ and $\beta_{c}$ denote the weights of the clustering and separation losses, respectively, for an anchor class *c*. We use an adaptive weighting of the losses, as described in Section 4.

### 3.3. Multitask Learning

Following contrastive pre-training, we add two task classifiers: a margin delineation classifier and a diagnosis classifier (see Figure 1). The margin delineation classifier labels pixels as lesion or healthy. For margin delineation, benign and malignant lesions are grouped in a general “lesion” class. The diagnosis classifier labels pixels as benign or malignant. During training, we group healthy samples with benign samples for the diagnosis task.

The total loss for the multitask learning phase ($L_{MT}$) is as follows:

(4) $$L_{MT}=CE_{\mathrm{diag}}+CE_{\mathrm{delin}}+L_{\mathrm{reg}}$$

where $CE_{diag}$ and $CE_{delin}$ are the cross-entropy losses for the diagnosis and delineation classifiers, respectively. $L_{reg}$ is a regularization loss that ensures consistency between the two classifiers. Specifically, the regularization loss is a cross-entropy loss that penalizes the network for labeling malignant samples differently. For example, we penalize cases when the margin delineation classifier labels a malignant sample as “healthy”, but the diagnosis classifier labels the same malignant sample as “malignant”). Similarly, healthy tissue samples cannot simultaneously be cancerous, so any such predictions are penalized by the regularization loss.

### 3.4. Biological Basis and Imaging System

Though there are many types of cancers, Hanahan and Weinberg [31] initially described the development of cancer in 2000 as a collection of six basic cellular changes that underlie the development of most cancers. These six basic changes, or “hallmarks of cancer”, were updated in 2011 to include changes in cellular metabolism as a potential additional hallmark [32]. In 2022, Hanahan [33] adopted “deregulating cellular metabolism” as an additional hallmark. As metabolic cofactors involved in oxidative phosphorylation and glycolysis [34,35,36], NADH and FAD have been investigated as potential autofluorescence biomarkers for pre-cancer or cancer by several studies (e.g., [37,38,39]).

Specifically for oral cancer, Sethupathi et al. [40] induced carcinogenesis using DMBA in a hamster model and measured the NADH and FAD autofluorescence. The autofluorescence values were used to calculate the redox ratio [40]. The redox ratio was compared through the development of cancer, showing that the redox ratio decreases as the induced lesions advance from normal to dysplasia to cancer [40]. In human cell lines, Shah et al. [41] show increased NADH and FAD autofluorescence in cancerous cell lines compared to normal cells with *p*-values < 0.05. Based on such insights, the imaging system excites tissue in a single excitation band and collects the response from endogenous fluorophores to quantify the optical characteristics of the tissue.

The collected data capture both the intensity of the response, as well as the temporal dynamics as the fluorescence decays over time. The tissue autofluorescence is collected in three emission bands (channels) corresponding to collagen, reduced *nicotinamide adenine dinucleotide* (NADH), and *flavin adenine dinucleotide* (FAD). The optical properties of collagen describe the structural characteristics of the tissue, which have been shown to change with neoplastic transformation (e.g., extracellular matrix remodeling) [42]. NADH and FAD autofluorescence can be used to calculate the optical redox ratio, which describes the metabolic state of the tissue [43,44,45,46]. A lower redox ratio is associated with an increase in cellular metabolism, which is an indicator of malignant transformation [47]. In head and neck cancers, changes in tissue concentration of NADH and FAD have been demonstrated [41,48,49]. The lifetime of the collected autofluorescence provides additional information about the tissue microenvironment, including protein binding changes associated with cancer development [37,50].

Further design details for the imaging system used for data collection have been previously reported by Cheng et al. [51]. The temporal resolution is 0.25 ns and the *field of view* (FOV) is a circular region with an approximate diameter of 1 cm. The system is a single-excitation system, with a 355 nm excitation laser that exposes the tissue to only 2.8 mJ of energy. The amount of energy deposited to the tissue is within the *maximum permissible exposure* (MPE) of 29.8 mJ set by the *American National Standards Institute* (ANSI) [52]. The pulse width of the excitation laser is 1ns. The resulting autofluorescence emission is captured in three emission bands. The first band is 390 ± 20 nm to capture collagen autofluorescence. The second band is 452 ± 22.5 nm to capture NADH autofluorescence. The final emission band is >500 nm to capture FAD autofluorescence. The time required to image is less than 3 s for each image. For each patient, we collect an image of the center of the oral lesion and an image of normal-appearing tissue. We non-invasively image each patient’s oral lesion before the biopsy. Following our imaging protocol, patients undergo biopsies that are used to generate a pathology diagnosis for our ground-truth labels. The raw data collected by the system is a biexponential-like fluorescence decay for each channel at each pixel location (see Figure 1F).

### 3.5. Data and Preprocessing Steps

Our dataset consists of 67 lesion images and 67 healthy images, with an image size of 160 × 160 pixels (approx. 3.4 million pixels). The lesion diagnoses are as follows: 33 benign, 5 dysplasia, and 29 *squamous cell carcinoma* (SCC). For the classification tasks, we combined dysplasia and SCC in the same group. Institutional review board approval for the study was provided by Hamad Medical Corporation (Doha, Qatar). A detailed breakdown of the lesion distribution by diagnosis and anatomical location is given in Table 1. Notably, the dataset contains imbalance at both the diagnosis level (only five cases are diagnosed as dysplasia) and at the anatomical location level (over half of the lesions are located in the mucosa or tongue regions).

Cross-validation folds for a single trial were generated by randomly splitting the images into 10 folds, preserving class balance as much as possible within each fold. Within each run, we used 7 folds for the train set, 2 folds for the development set, and 1 fold for the test set. We conducted ten runs for each trial, ensuring that each fold was used as the testing set once per trial. We refer to the average results of all ten runs as a trial. We repeated the random splits 10 times, resulting in a total of 10 trials (10 trials × 10 runs per trial = 100 models).

Each image was pre-processed using median filtering with a sliding window across pixels to increase the SNR. Median filtering with a sliding window increased the SNR but retained the original image size (160 × 160 pixels). After median filtering, pixels with low SNR were masked to exclude from training. At the pixel level, the first preprocessing step involved signal inversion for each decay. Next, each channel was zero-padded to 300 samples (total network input of 3 channels × 300 samples per channel = 900-dimensional input). A calibration factor was applied to each channel to adjust for day-to-day variations in the system. Finally, each pixel signal (concatenated decays of the three channels) was normalized to sum to 100 to adjust for the different gains used during data collection. Deconvolution of the instrument response from the raw fluorescence decay, as typically performed in FLIM data analysis, was not needed in the proposed framework. An example of a pre-processed 3-channel decay, ready for input to the network, is shown in Figure 1 (example pixel).

### 3.6. Implementation

**Figure 2:**
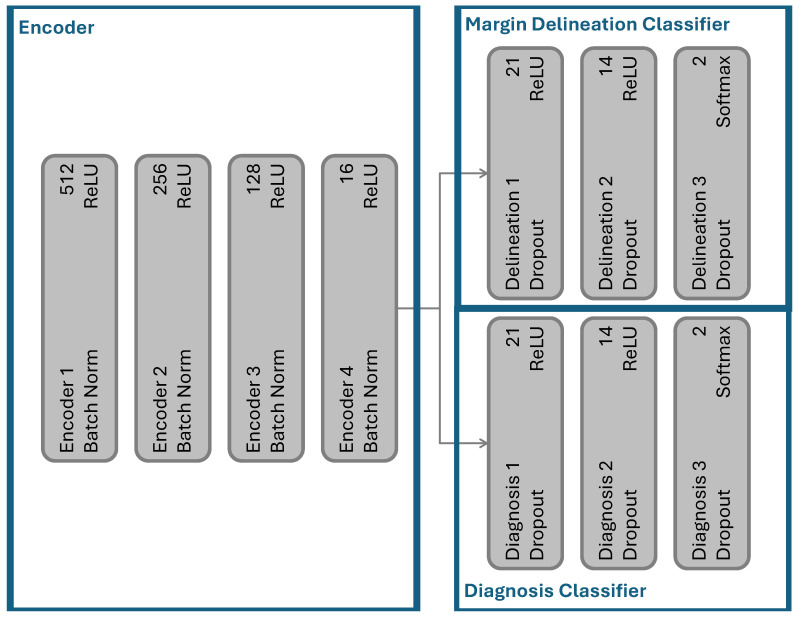
The network structure of the proposed architecture.

The specific structures for the encoder and classifiers are given in Figure 2. Each layer has three components: a fully connected layer, internal regularization, and activation. We used batch normalization in the encoder for regularization. Batch normalization has been reported to smooth the training surface [53], speed training [53,54], and improve generalization [53,54]. As described in Section 4, we also found that batch normalization stabilizes the separation terms in our contrastive loss. We trained the encoder with the contrastive loss given in Equation (1). We applied the loss to the fourth encoder layer after batch normalization and before the *rectified linear unit* (ReLU) activation. We optimized with Adam [55] (learning rate of 1 × 10^−5^, batch size of 512). For additional stabilization, we used gradient clipping at 0.25. We trained the encoder for 10 epochs and picked the best model based on the development set. After the encoder was trained, we added the task classifiers.

In the task classifiers, we used dropout for regularization (p=0.5). By randomly removing nodes during training, dropout resulted in an ensemble-like model and reduced overfitting [56]. We optimized Equation (4) using Adam [55] (learning rate of 1 × 10^−5^, batch size 256). The number of trainable parameters was 630,814 and the number of *floating point operations* (FLOPS) was 0.00126 G. The model was trained for an additional five epochs, with the best model selected based on the development set’s performance. Due to class imbalance (heavily biased toward the healthy class), we used sample weights calculated based on the training distribution, employing the *class weight* from *sci-kit learn* [57].

The task classifiers are trained on the pixel level. However, we report results at the image level. To determine the image-level label, we aggregate the labels of each pixel from an image and use a 50% threshold, taking the majority label of the image’s pixels.

## 4. Results

The experiments in this section are organized as follows: contrastive loss function validation (Section 4.1), contrastive encoder training (Section 4.2), full model evaluation (Section 4.3 and Section 4.4), and analysis of the contributions of the model’s components (Section 4.5).

### 4.1. Loss Function Validation

**Figure 3:**
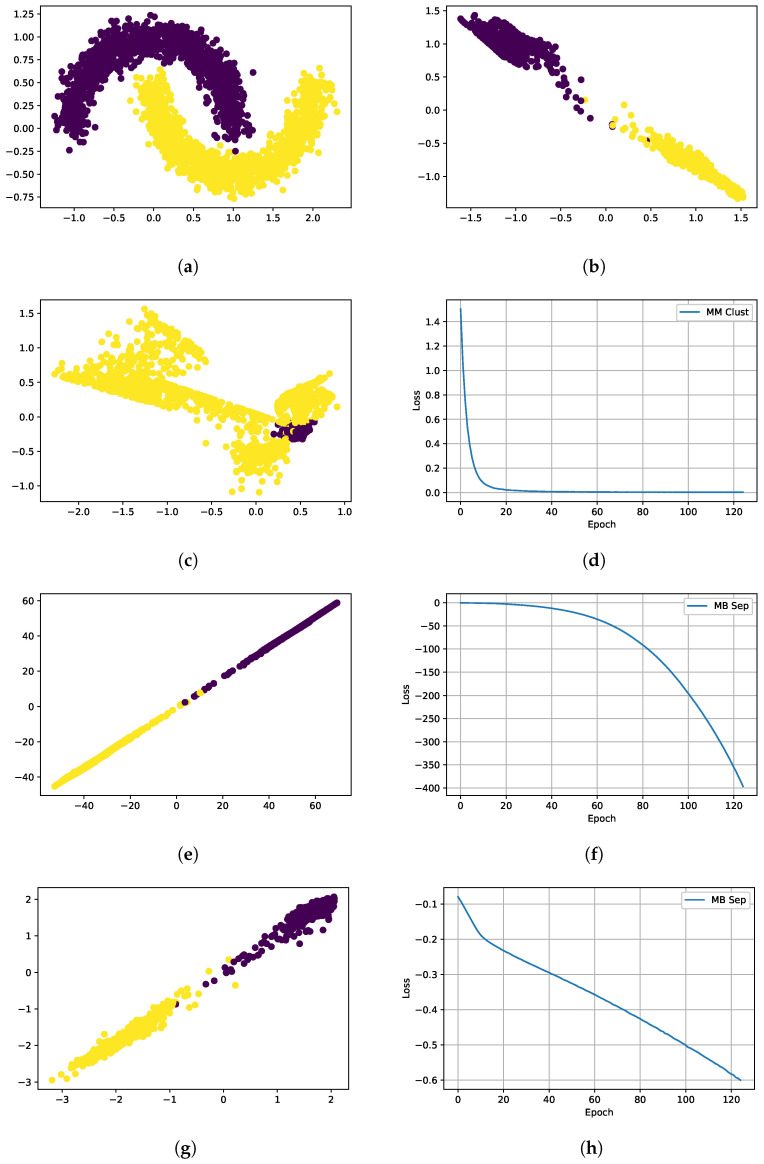
Contrastive training with synthetic data. The clustering and separation losses work together to separate the classes. Each component functions alone as expected when batch normalization is used. (**a**) Synthetic 2 class training data. (**b**) After: Clust. and Sep. losses with batch norm. (**c**) Single class clustering result. (**d**) Single class clustering loss. (**e**) Sep. loss collapse w/o batch norm. (**f**) Sep. loss training curve w/o batch norm. (**g**) Sep. loss only training w/batch norm. (**h**) Separation loss curve with batch norm.

We first used a toy example to understand how our loss function affected clustering (see Figure 3). As we developed our method, the synthetic data functioned as a simplistic simulation, where we had better control over the difficulty of the clustering task compared with our actual data. In the actual data, we could not control noise levels or the distances between classes. By gradually increasing the difficulty in synthetic data (starting with two classes and moving to three classes), we learned to adaptively weight the loss components and use batch normalization to achieve better clustering separation and keep the loss components from diverging. We used the *make moons* tool from *sci-kit learn* [57] to generate synthetic data and used dense layers with the contrastive loss function. Starting with a two-class example (see Figure 3a), we can show that the loss function clusters each class together, away from the other class (Figure 3b). Figure 3 also shows the results of each component of the loss function. As expected, the clustering component effectively clustered a single class, as seen in Figure 3c,d. The separation loss, however, presented more interesting behavior. Without batch normalization, the separation loss diverged (Figure 3e,f). If we added a batch normalization layer, the separation loss decreased in a controlled manner, and the classes separated as expected (Figure 3g,h).

**Figure 4:**
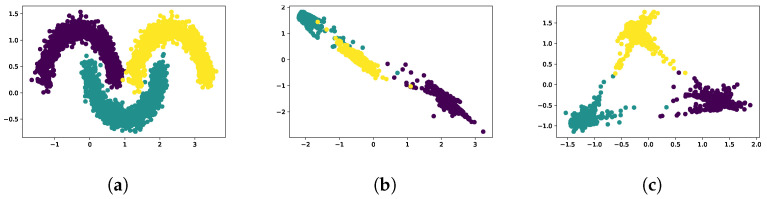
Contrastive training with three classes. All three classes were separable after using the adaptive weighting scheme. (**a**) Synthetic 3 class training data. (**b**) Final result using clustering and separation losses without adaptive weighting. (**c**) Final result using clustering and separation losses with adaptive weighting.

**Figure 5:**
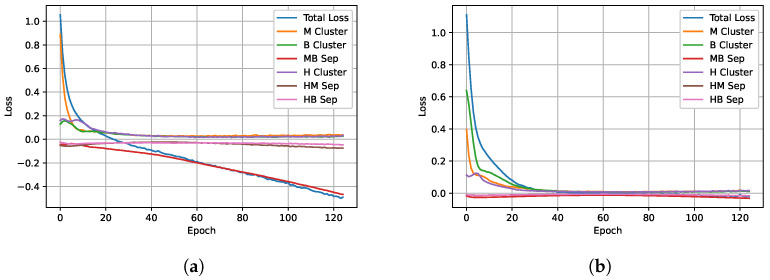
Loss functions for contrastive training with three classes. Each component of the loss function is more stable with the adaptive weighting. (**a**) Training losses without adaptive weighting. (**b**) Training losses with adaptive weighting.

Next, we introduced a third class of synthetic data to determine if our loss function could effectively separate three classes (Figure 4a). As shown in Figure 4b, only two classes separated well when the loss components had a static weight set at the beginning of the training. The training losses in Figure 5a show that one of the separation loss components began to dominate, resulting in good separation between the purple class and the other two. However, because the model received significant rewards for distancing the purple class from the other two, the separation of the other two classes was ignored. To balance the losses, we adaptively weighted the separation losses ($\beta_{c}$ from Equation (3)) inversely proportional to the distance between the class means:

(5) $$\beta_{c}=\frac{1}{dist_{A,Neg}}$$

where $dist_{A,Neg}$ is the squared distance between the means of the anchor class and the negative class. The weights are normalized such that they sum to one. The resulting weighting for a loss component is larger if two classes are poorly separated, but decreases as the separation increases. As shown in Figure 4c, the adaptive weighting strategy results in separated classes with nearly equidistant cluster centers. In addition, the loss curves in Figure 5b show that none of the separation loss components dominates.

### 4.2. maFLIM Contrastive Pre-Training

To visualize the progress of contrastive pre-training on maFLIM data, we used the encoder and added a two-dimensional layer on which the contrastive loss operated (i.e., the dimension of the bottleneck representation was reduced from 16 to 2 for visualization). We also monitored the silhouette score [58] of the two-dimensional representation using the *sci-kit learn* implementation [57]. The silhouette score quantified the clustering performance and can be calculated as follows:

(6) $$S=\frac{B-A}{max(A,B)}$$

where *A* is the mean distance between a sample and all other points in the same class and *B* is the mean distance between a sample and all other points in the next nearest cluster. The range of the silhouette score is from −1 to 1. A negative value indicates wrong assignments and a large positive value indicates high intra-class clustering and high inter-class separation. We use the Euclidean distance as the distance metric, although any distance metric may be used.

**Figure 6:**
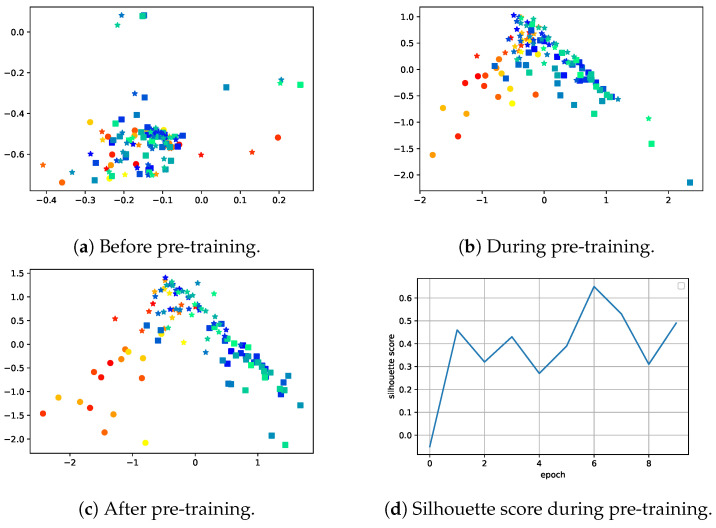
Two-dimensional (2D) contrastive feature space representation and silhouette scores during pre-training. The shapes represent each class. The stars represent healthy samples. The circles represent malignant samples. The squares represent benign samples. The cool colors represent samples from patients with benign lesions, while the warm colors represent samples from patients with malignant lesions. The shades of warm and cool colors help differentiate between tightly clustered patients, but have no additional meaning.

Figure 6 shows the 2D representations through contrastive training with the corresponding silhouette score. The warm colors (yellows and oranges) represent the mean embedding from patients with malignant lesions. The cool colors (blues and greens) represent the mean embedding from patients with benign lesions. The different shades in the colors represent different patients. The circles, squares, and stars represent the mean embedding from malignant, benign, and healthy images, respectively. For example, a patient with a malignant lesion will have two points plotted on the graph: an orange or yellow circle and a star in the same color.

Using these two-dimensional plots, we found that the weights for Equation (2) could cause the representation space to collapse, resulting in all points clustering together, regardless of their class (i.e., the axes in the plots would span very little space). To prevent the space from collapsing, we implemented an adaptive weighting scheme for the $\alpha_{c}$ values based on the silhouette score. As the silhouette score increased, indicating good clustering, we gradually decreased the $\alpha_{c}$ weight. The adaptive weighting enabled the data to cluster quickly initially, but avoided instability due to dominance of the clustering term near the end of the contrastive pre-training.

Figure 6a shows the representation before contrastive training, where there was no separation between classes in two dimensions and the silhouette score was slightly negative. As training progressed (Figure 6b), the 2D representation began to show distinct class clustering and the silhouette score increased. As shown in Figure 6c, after training, the three classes were well clustered and the silhouette score neared 0.9. The contrastive pre-training step successfully groups each class and creates distance between different classes. In addition, Figure 6 shows that the silhouette score quantitatively describes the quality of the clusters visually depicted on the two-dimensional graphs. In the experiments that follow, we used the training set silhouette score to monitor training progress, as the 16-dimensional embedding in the full model cannot be directly visualized.

### 4.3. Diagnosis

We report the results of the full model (contrastive encoder plus multitask classification) on the diagnosis task in Table 2. Although the diagnosis classifier was trained using benign, malignant, and healthy samples, the evaluation was conducted only on the lesion samples. We compare the full model to four baselines: SVM with SFS, SVM with L1 regularization, an autoencoder model, and a multitask model. We have previously reported results with the SVM and autoencoder methods [13], while the multitask baseline enabled a more direct evaluation of the contrastive pre-training step. The multitask baseline refers to the full model without the contrastive pre-training step. On average, adding the contrastive pre-training step improved the test performance by 2.33%, emphasizing the effectiveness of our patient normalization step. In comparison to the autoencoder, the full model showed an average improvement of 1.46%. In addition, the full model’s increase of 8.34% in specificity represents a statistically significant improvement over the autoencoder method, as determined by a paired, one-tailed *t*-test (*p* = 0.0070). We note that the full model achieved better performance despite training on fewer lesion images than the autoencoder method, which used training images from two domains. When we compare this approach with the single-task SVM baseline proposed in Caughlin et al. [13], we observe improvements ranging from 2.75% to 4.87%. Using the average of sensitivity and specificity as a metric, a one-tailed, paired *t*-test shows a significant improvement over both SVM methods. The *p*-value for the SVM SFS method was 0.0261, while the *p*-value for the SVM L1 method was 0.0452. Although the full model outperformed all single-task baselines, we cannot attribute the increase in performance solely to the multitask framework as the multitask-only baseline does not perform better than the autoencoder baseline. Rather, the combination of patient normalization and multitask training achieves the best performance. We present an ablation study of the model components for the diagnosis task in Table 3.

### 4.4. Margin Delineation

We evaluate the margin delineation classifier for two clinical use cases. The first case is margin delineation between malignant and healthy tissue. This classifier would be used when a patient has a known malignant lesion. The classifier would aid resection by showing the clinician how much tissue to remove. The second case involves margin delineation between a lesion (regardless of diagnosis) and healthy tissue. This classifier would be used when a patient presents a suspicious lesion but has not undergone a biopsy or received a confirmed diagnosis. By removing the entire lesion and ensuring the boundary is also excised, the patient may not need to return for further resection, even if the diagnosis is malignant. We anticipate that this task will be more challenging due to the difficulty in distinguishing between benign and healthy tissue samples.

As in the diagnosis case, the contrastive pre-training plus multitask framework (full model) outperforms the multitask-only network by 0.6% to 3.4%. In addition, the full model has good performance on both types of margin delineation, reducing the performance gap between use cases from 6.17% to 3.37%. Although margin delineation is an auxiliary task in our framework, Table 4 shows that the classifier achieves good performance on margin delineation as well.

### 4.5. Analysis of the Model’s Components

The performance of the network with various components removed (ablation study) is shown in Table 3 and Table 4. Comparing rows 1 and 4 for each task, the tables show that the contrastive pre-training step improves average performance across all tasks. The consistency loss improves average diagnosis performance, our primary task, by 0.92% (see columns 2 and 3 in rows 4 and 5 of Table 3). When compared to single-task training, multitask learning produced similar results on the diagnosis task. However, the performance is more evenly balanced in terms of sensitivity and specificity in the multitask setting. Finally, using adaptive clustering weights in the contrastive step improves performance by 2.21% (columns 1 and 4 in rows 1 and 2 in Table 3).

## 5. Conclusions

Our multitask learning framework, enhanced with contrastive pre-training, improves diagnosis performance across all baselines and also delivers good performance on margin delineation as an auxiliary task. The contrastive step helps group the classes compactly together, away from the other classes. This strategy aids patient normalization, without requiring comparison to a reference sample for each patient at test time, and provides a favorable initialization for multitask training. The multitask classifiers allow the margin delineation task and diagnosis task training to benefit each other, further regularizing the network and improving generalization.

A limitation of our work involves the size of the dataset. However, we included several strategies to reduce potential problems with small data. Our pixel-level training process resulted in many more samples for training. Due to the novel data format, our models were trained at the pixel level rather than the image level. Since each image contained 160 × 160 pixels and we took images of each patient’s normal and lesion tissues, we had 160 × 160 × 2 = 51,200 pixels (samples) per patient before preprocessing. While we did exclude some of these pixels during preprocessing based on SNR, using 51,200 pixels per each of the 67 patients led to over 3.4 million pixels/samples. We note that our total patient count is comparable to related research using fluorescence lifetime imaging systems (e.g., the work by Marsden et al. [6], with 53 patients compared to our 67 patients). In the related field of fluorescence lifetime imaging microscopy, several studies have used similar sample sizes. In a lung cancer application of fluorescence lifetime imaging microscopy, Wang et al. included a total of 31 patients in their study [59]. Ji et al. used a database of 71 patients with fluorescence lifetime imaging microscopy data for a machine learning application in cervical cancer risk [60].

In addition, we carefully designed our machine learning models to reduce bias in the predictions given the smaller sample sizes in our database. In our baselines and proposed methods, we used regularization methods to reduce overfitting and improve generalization. In the SVM baseline, we used L1 regularization. L1 regularization encourages some coefficients to go to zero, creating a simpler decision function that focuses on the most relevant features. In our proposed method, we implemented multitask learning as a form of regularization. In multitask learning, the network was constrained to find a solution that satisfied both tasks, leading to more robust representations and classifiers. We also limited the network size to reduce the likelihood of overfitting. Our feature extractor consists of only four layers, and the classifier has just three layers, including the final two-node classification layer. Finally, we meticulously applied a 10-fold cross-validation scheme and repeated the experiments for 10 full trials, ensuring that our results are an average over 100 different models.

Although our analysis only considers regularization and patient normalization through our contrastive pre-training, other automated medical tasks may benefit from similar approaches. For example, as datasets grow larger, our approach naturally extends to more specific classes. We may be able to extend our multitask setting to consider different grades of pre-cancer or to accommodate separate classes for different types of benign lesions. For example, given the adequate dataset size, our approach could be easily modified to provide a more specific diagnosis by adding additional clusters during pre-training that separate SCC, mild, moderate, and severe dysplasia. Following pre-training, the multitask learning framework could be modified to include additional outputs in the diagnosis classifier to accommodate the more specific classes of dysplasia and cancer. Using the contrastive approach may also improve domain generalization. Domain generalization is required for clinical translation when a trained model is deployed in a new location. Taking inspiration from the contrastive learning work in the natural image domain, (e.g., Kim et al. [61]), we would like to extend our work to the multi-center setting, where multiple small datasets from separate imaging centers need to be merged. In this setting, the clustering losses in the contrastive pre-training step would encourage samples from the same class to group compactly, regardless of domain shifts due to differences in imaging centers.

## Figures and Tables

**Table 1:** Lesion distribution in the database.

Location	Benign	Dysplasia	SCC
Mucosa	10	3	9
Floor of Mouth	2	0	1
Gingiva	0	2	3
Lip	10	0	2
Mandible	0	0	1
Palate	1	0	0
Maxilla	0	0	1
Retromolar	1	0	0
Tongue	9	0	12
Total	33	5	29

**Table 2:** **Diagnosis results.** Full: contrastive pre-training with adaptive weights followed by multitask learning with consistency loss. Multitask: no contrastive pre-training. AE: autoencoder and a single-task classifier method. SVM SFS: support vector machine with sequential feature selection. SVM L1: support vector machine with L1 regularization.

Model	Sens.	Spec.	Avg.	Prec.	F1	Acc.
Full	82.08	75.92	79.00	76.92	78.07	78.81
Multitask	77.92	75.42	76.67	76.02	74.77	76.50
AE	87.50	67.58	77.54	76.25	79.80	77.62
SVM SFS	81.00	67.25	74.13	73.82	74.92	74.05
SVM L1	79.17	73.33	76.25	78.10	75.92	76.36

**Table 3:** **Ablation results: diagnosis task**. PT: pre-train. ACW: adaptive clustering weighting. CL: consistency loss. MT: multitask learning. Avg.: average of sensitivity and specificity.

Task	Clust. PT	Sep. PT	ACW	CL	MT	Sens.	Spec.	Avg.
	✓	✓	✓	✓	✓	82.08	75.92	79.00
	✓	✓		✓	✓	77.58	76.00	76.79
	✓	✓	✓			74.25	79.58	76.92
Diag.					✓	76.50	75.00	75.75
				✓	✓	77.92	75.42	76.67
	✓		✓	✓	✓	83.00	73.58	78.29
		✓		✓	✓	79.50	75.50	77.50
						71.92	81.50	76.71

**Table 4:** **Ablation results: margin delineation task**. PT: pre-train. ACW: adaptive clustering weighting. CL: consistency loss. MT: multitask learning. Avg.: average of sensitivity and specificity.

Task	Clust. PT	Sep. PT	ACW	CL	MT	Sens.	Spec.	Avg.
	✓	✓	✓	✓	✓	91.83	79.31	85.57
	✓	✓		✓	✓	88.33	79.19	83.76
	✓	✓	✓			89.33	75.64	82.49
M-H					✓	88.33	82.31	85.32
				✓	✓	84.83	85.10	84.97
	✓		✓	✓	✓	91.33	79.76	85.55
		✓		✓	✓	90.50	80.22	85.36
						89.83	80.98	85.41
	✓	✓	✓	✓	✓	85.10	79.31	82.20
	✓	✓		✓	✓	79.00	79.22	79.11
	✓	✓	✓			84.02	75.64	79.83
L-H					✓	75.36	82.31	78.83
				✓	✓	72.53	85.07	78.80
	✓		✓	✓	✓	80.72	79.76	80.24
		✓		✓	✓	80.24	80.22	80.23
						80.76	80.98	80.87

## Data Availability

Please contact Javier Jo (javierjo@ou.edu) regarding data access. As the data used in this study were obtained from human subjects, data are not publicly available.
